# Bamboo-Dominated Forests of the Southwest Amazon: Detection, Spatial Extent, Life Cycle Length and Flowering Waves

**DOI:** 10.1371/journal.pone.0054852

**Published:** 2013-01-24

**Authors:** Anelena L. de Carvalho, Bruce W. Nelson, Milton C. Bianchini, Daniela Plagnol, Tatiana M. Kuplich, Douglas C. Daly

**Affiliations:** 1 Graduate Program in Forest Science, National Institute for Amazon Research, Cruzeiro do Sul, Brazil; 2 Ecology Department, National Institute for Amazon Research, Manaus, Brazil; 3 Graduate Program in Ecology, Instituto de Criminalística do Amazonas - IC/Departamento de Polícia Técnico Científica – DPTC/Polícia Civil do Estado do Amazonas – PCAM, National Institute for Amazon Research, Manaus, Brazil; 4 Ecology Department, Graduate Program in Ecology, National Institute for Amazon Research, Manaus, Brazil; 5 National Institute for Space Research, Southern Region (INPE/CRS), Santa Maria, Brazil; 6 The New York Botanical Garden, Bronx, New York, United States of America; University of Western Australia, Australia

## Abstract

We map the extent, infer the life-cycle length and describe spatial and temporal patterns of flowering of sarmentose bamboos (*Guadua* spp) in upland forests of the southwest Amazon. We first examine the spectra and the spectral separation of forests with different bamboo life stages. False-color composites from orbital sensors going back to 1975 are capable of distinguishing life stages. These woody bamboos flower produce massive quantities of seeds and then die. Life stage is synchronized, forming a single cohort within each population. Bamboo dominates at least 161,500 km^2^ of forest, coincident with an area of recent or ongoing tectonic uplift, rapid mechanical erosion and poorly drained soils rich in exchangeable cations. Each bamboo population is confined to a single spatially continuous patch or to a core patch with small outliers. Using spatial congruence between pairs of mature-stage maps from different years, we estimate an average life cycle of 27–28 y. It is now possible to predict exactly where and approximately when new bamboo mortality events will occur. We also map 74 bamboo populations that flowered between 2001 and 2008 over the entire domain of bamboo-dominated forest. Population size averaged 330 km^2^. Flowering events of these populations are temporally and/or spatially separated, restricting or preventing gene exchange. Nonetheless, adjacent populations flower closer in time than expected by chance, forming flowering waves. This may be a consequence of allochronic divergence from fewer ancestral populations and suggests a long history of widespread bamboo in the southwest Amazon.

## Introduction

The woody bamboos *Guadua weberbaueri* Pilger and *G. sarcocarpa* Londoño & Peterson dominate forests over a large area of the southwest Amazon. They are sarmentose, i.e. basally erect but distally climbing, supporting themselves on trees by means of recurved spines [1]. Flowering, fruiting and subsequent mortality all occur just once in the last year of a multi-decade life cycle. All or most individuals in a population belong to a single reproductively synchronized cohort. As in some other tropical and subtropical forests with upper canopy dominated by bamboos, the adult stage [2], [3] and the post-flowering mortality stage [4], [5] can be detected in images from orbital sensors with optical bands, such as Landsat Thematic Mapper (TM) and the Moderate Resolution Imaging Spectroradiometer (MODIS). Forest with mature bamboo is, however, spectrally similar to secondary forest in swidden agriculture plots [6]. The largely intact forests of the southwest Amazon afford a unique opportunity to map almost the entire primitive extent of a bamboo-dominated tropical forest. With a time series of annual satellite images, one can also determine the precise extent and flowering time of each bamboo population. Hereafter we refer to all sarmentose *Guadua* spp. as “bamboo”.

**Figure 1:**
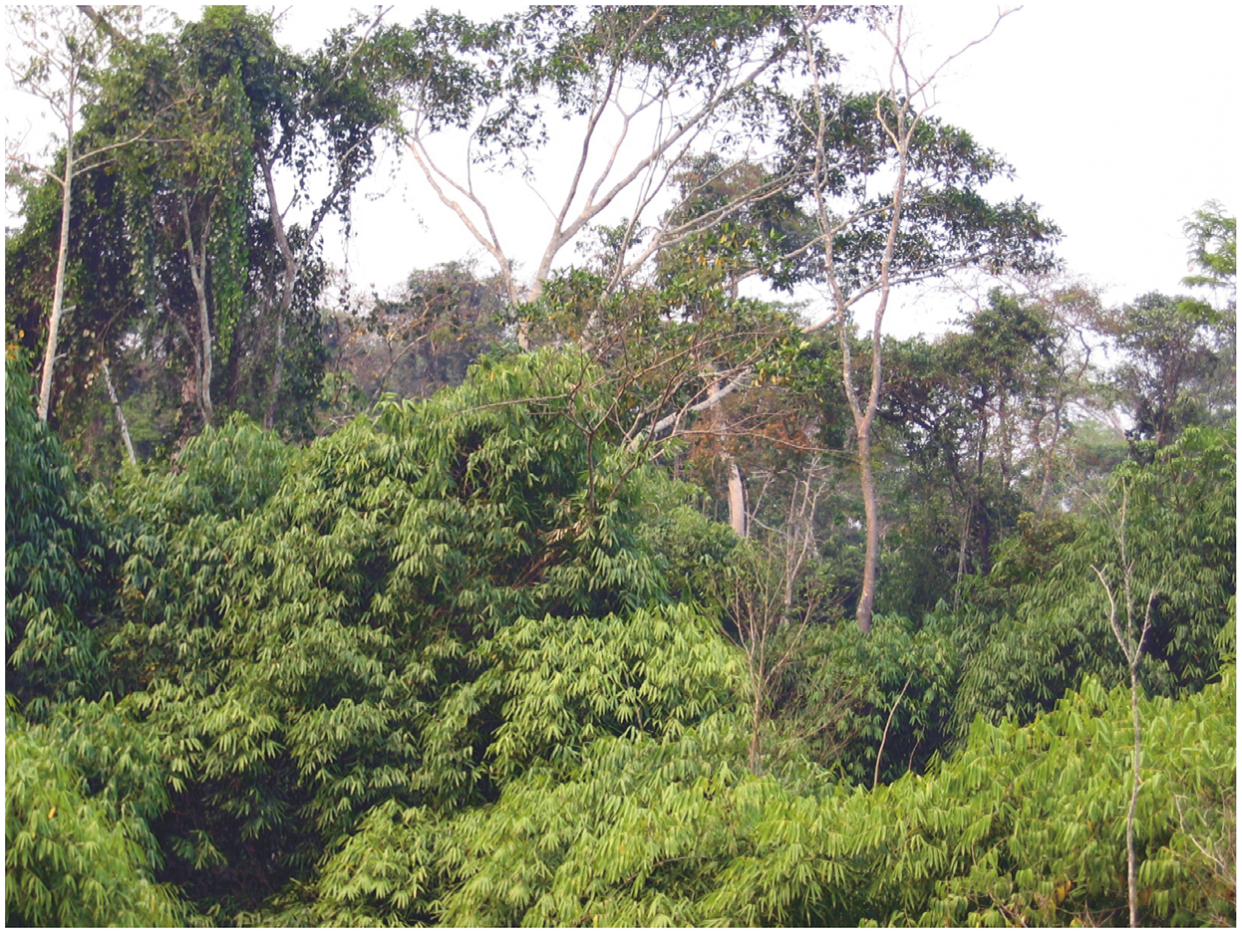
Forest canopy dominated by mature *Guadua sarcocarpa*. Sixteen year-old cohort of climbing bamboo forms a dense carpet weighing heavily on mid-canopy trees. Upper canopy trees are widely spaced. Location is 8° 58′ 56′′ S, 68° 42′ 34′′ W, date September 18, 2004.

Previous studies examined differences in structure and composition between forests with and without bamboo in the southwest Amazon [1], [7], [8] and the relationship between bamboo and forest disturbance [1], [9], [10]. When compared to a nearby forest without bamboo on the same soil type, bamboo presence reduced live aboveground forest biomass by more than 40% for all stems over 2.5 cm diameter at breast height (DBH). Bamboo disturbs the forest by causing mechanical damage to trees and saplings [1], creating large canopy gaps (Figure 1) that are colonized by both bamboo and fast-growing trees of low wood density [7], [8]. Understory trees show evidence of repeated stem breakage. However, large trees are the size class most affected and the mechanism by which bamboo causes their rarefaction is still unclear. Palms are the life form that most suffers in the presence of bamboo, with *Euterpe precatoria* Martius and *Iriartea deltoidea* Ruiz & Pavon almost completely eliminated. Perhaps because of this background disturbance, the flora and fauna that do coexist with bamboo are highly resilient after ground fire [10]. Disturbance by bamboo is reduced for several years after an adult cohort dies and decomposes. But by 10–13 y of age, the succeeding juvenile cohort successfully retakes the canopy spaces relinquished by death of the previous adult generation. In a cohort of 10 y age, A.C. Oliveira found 860+/−350 (average +/−1 SD) bamboo culms ha^−1^>2.5 cm DBH [8]. From 10 to 13 y of age, culm density nearly doubled and basal area increased almost three-fold as this cohort reached the upper forest canopy [9], becoming clearly visible in Landsat images.

In this paper we use images from three orbital sensors to map the full extent of bamboo and of individual bamboo populations. The latter are detected at the post-reproductive stage. Image interpretation is supported by the known locations and dates of five mass flowering events, by field checking of bamboo life stages and two over-flights. Specific objectives are:

1. Describe the spectral patterns and spectral separation of forests having bamboo at three stages: live mature cohort, dead post-flowering cohort and juvenile cohort confined to the understory (or bamboo absent from the upper canopy);
2. Using false-color composites of bands from optical orbital sensors which discriminate forests with different bamboo life stages, map the extent of southwest Amazonia’s bamboo-dominated forests;
3. Relate bamboo presence/absence to substrate characteristics, including fertility, erosion and tectonic uplift;
4. Estimate the length of the monocarpic life cycle;
5. Determine whether flowering times of neighboring populations are non-random, forming flowering waves, by examining the spatial and temporal patterns of populations flowering across the entire range of bamboo-dominated forest;
6. In the light of these patterns, discuss the likelihood that pre-modern human disturbance caused invasion of the area by bamboos.

## Results

### Spectral Patterns

**Figure 2:**
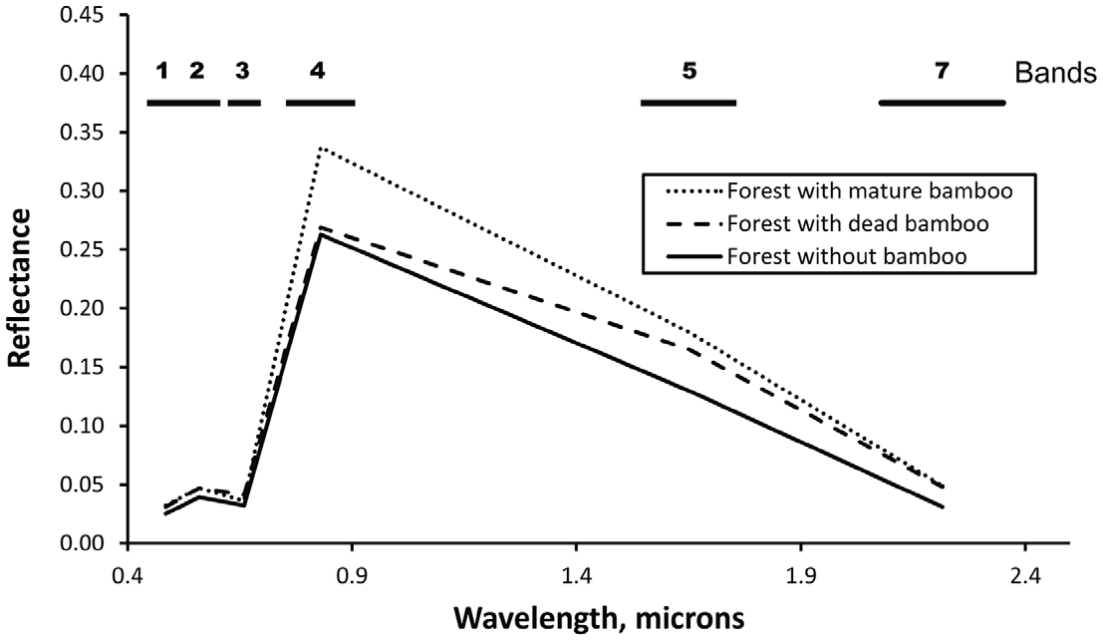
Reflectance spectra of three forest types. Landsat Thematic Mapper band numbers and positions are above the graph. Each vertex is an average of 20 spatially separate samples.

**Figure 3:**
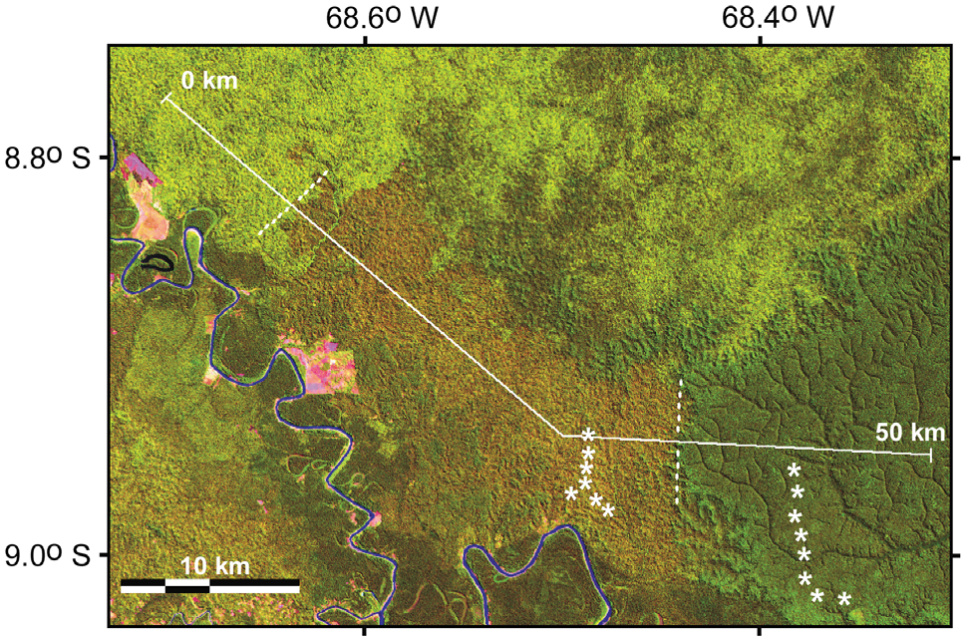
Three forest types in Landsat TM R-G-B composite of bands 5-4-3. Image acquired July 26, 1988. From left to right along 50 km transect indicated by solid white line: forest with mature bamboo (light green), with recently dead bamboo (rust brown) and bamboo-free forest on low infertile plateau (dark green). Border between mature and dead bamboo populations was not evident in a 1986 Landsat image (not shown) when both populations were alive and mature, exhibiting a light green color. Two sets of soil samples compared in Table 1 were collected at the white asterisks.

Figure 2 shows the spectra of forests with mature live bamboo, with recently dead bamboo and with no bamboo in the upper canopy, based on 20 samples of Landsat TM data in each of the three forest types, taken along a transect shown in Figure 3. All three canopy spectra follow the form typical of a green leaf. Bamboo presence, absence or mortality caused only minor differences. Therefore, all forest types are actually dominated by evergreen trees. This was confirmed by two over-flights, by field visits and forest inventories [8] near the area shown in Figure 3.

Nonetheless, linear discriminant analysis showed that the three spectra are distinct. TM band 4 (near-infrared) contributed the greatest amount of separation. For the 20 possible combinations of three TM bands, all but three successfully classified 100% of samples into their respective forest types using a leave-one-out jack-knife validation.

A false-color composite using TM bands 3-4-5 clearly distinguishes forests with mature bamboo, with recently dead bamboo and without bamboo in the upper canopy (Figure 3). These spatial differences were found to also occur over time, when examining this same type of false-color composite obtained before and after synchronous mortality, where five different bamboo populations were known to have flowered, based on herbarium specimens and field visits.

**Figure 4:**
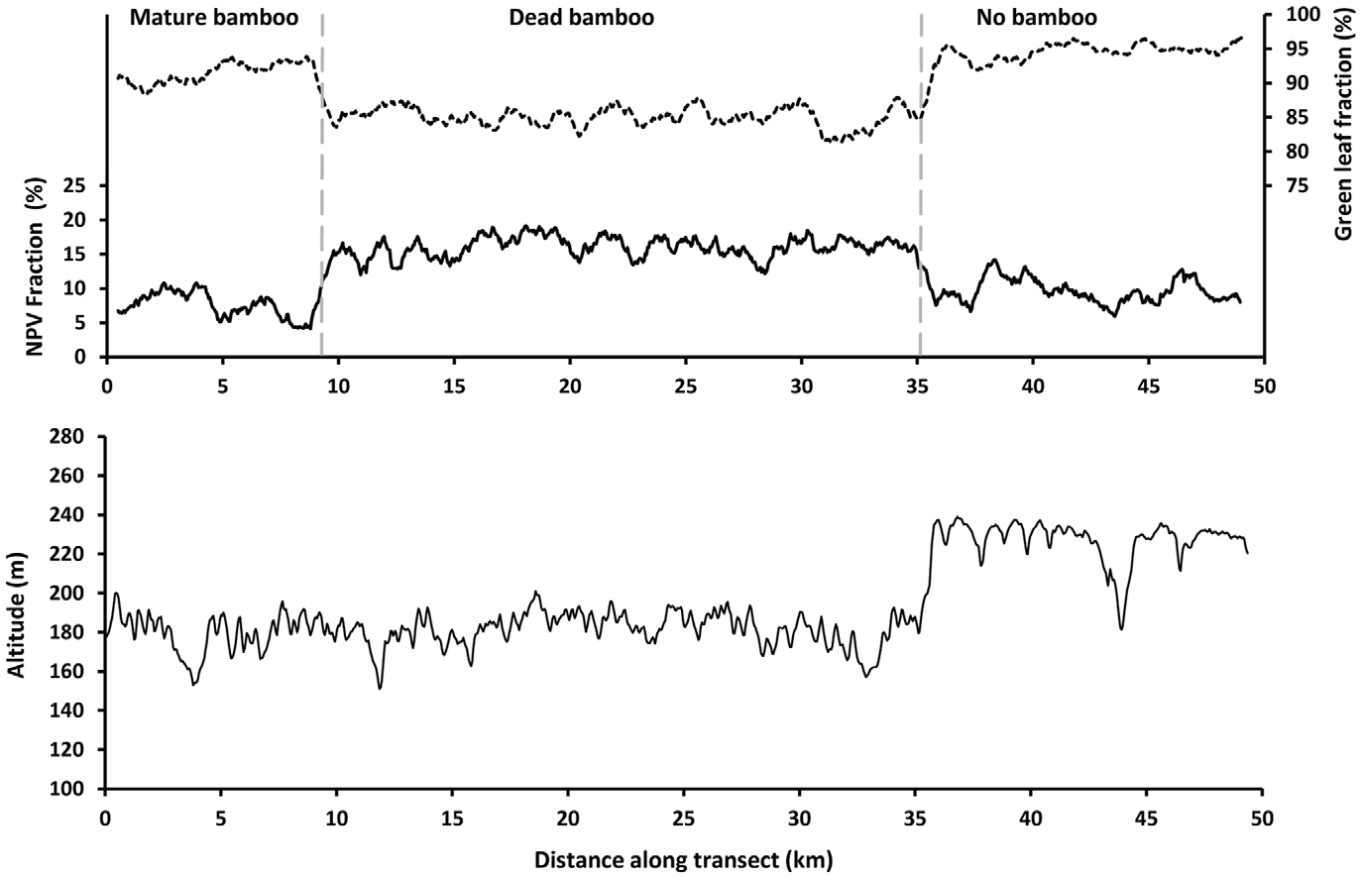
Greenness indicators and topography in the three forest types. Top graph shows green vegetation fraction (dashed trace) and non-photosynthetic vegetation fraction (solid trace) along the 50 km transect shown in Figure 3. Contacts between forest types are at the gray vertical dashed lines. Bottom graph is the same transect across a digital elevation model, contrasting topography of the hilly eroded landscape having fertile, poorly drained clay soil supporting bamboo-dominated forest and the plateau with infertile sandy latosol that excludes bamboo.

Green fraction and non-photosynthetic vegetation (NPV) fraction are transformed bands that describe the amount of green leaf and of bare branches, respectively, in the upper forest canopy. These were obtained by linear spectral unmixing of atmosphere-free reflectances of the six optical TM bands. Mean values of each of these canopy attributes were different between all pairs of the three forest types. Following the same transect crossing the three forest types shown in Figure 3, these transformed bands are traced in Figure 4. Because evergreen trees dominate the forest canopy, when bamboo dies the green leaf content of mixed pixels remains high. Crossing from a mature to a recently dead bamboo population, green vegetation fraction dropped only slightly, from 92% to 85%. The NPV fraction remained low, but doubled from 7% to 16%. A helicopter over-flight of a dying fruiting population of *Guadua sarcocarpa* revealed bare woody branches of senescing bamboo interspersed between leafy dicot trees. Dead bamboo branches rot and fall to the forest floor within two years.

A 150 km low-altitude over-flight of bamboo populations at the juvenile stage confirmed and extended previous studies of forest structure and composition [8], which found that the new cohort of bamboo derived from mast seeding is confined to the understory for about 8–10 y after seeding. Prior to attaining the upper canopy, the forest has many shade-rich gaps left by the former cohort. These gaps are not large, so they usually do not fill with dense secondary vegetation, making forest with juvenile bamboo indistinguishable from forest without bamboo in Landsat and MODIS images.

### Geographic Extent and Soils of Bamboo-dominated Forests

**Figure 5:**
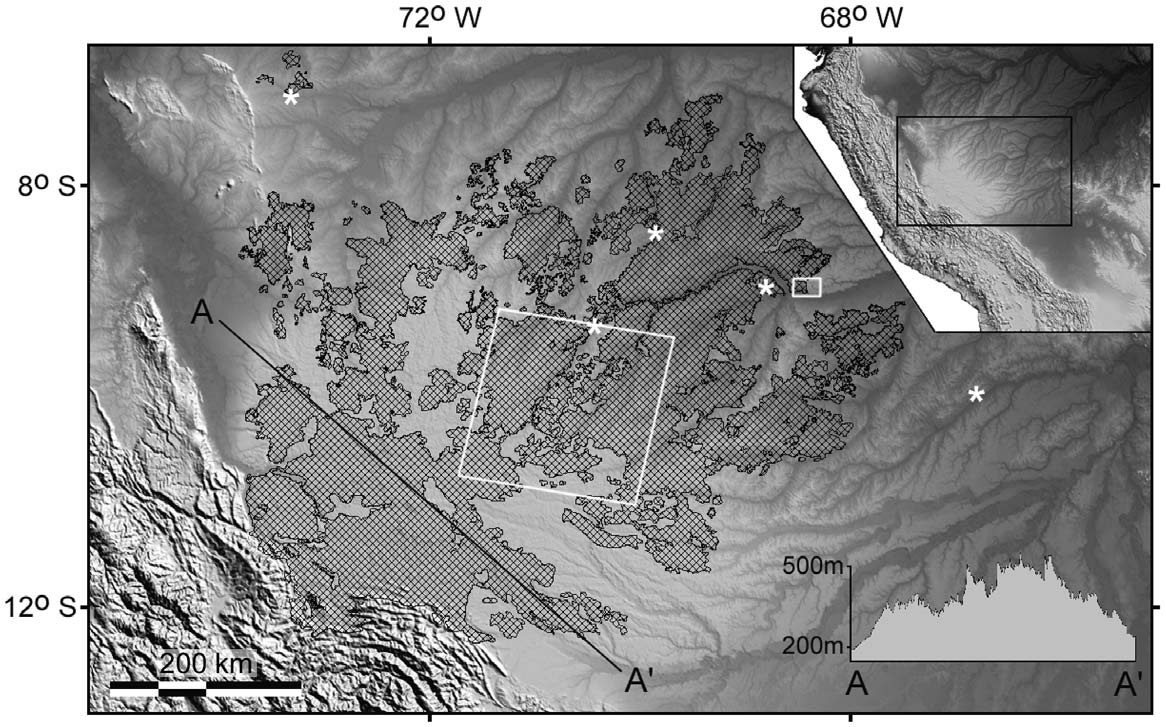
Full extent of southwest Amazon bamboo-dominated forests and study locations. Bamboo extent is based on visual interpretation of two Landsat Geocover mosaics and one MODIS mosaic. Base-map is a hill-shaded Digital Elevation Model from the Shuttle Radar Topography Mission, with gray tones scaled from 0–300 m altitude. Line A-A’ is a topographic profile across the Fitzcarrald Arch, also evident as higher terrain in the inset map. Soils and spectral patterns of forest types were compared at a topographically defined edge of bamboo forest in the small white rectangle, enlarged in Figure 3. Life-cycle study was conducted in the large white square. White asterisks are five sites of flowering or fruiting specimens of sarmentose *Guadua*, where Landsat images confirmed a temporal change in forest canopy spectral patterns from mature to post-reproduction life stage. Flowering specimens from west to east are: *Nelson 6026* (INPA), June 1995 (*Guadua sarcocarpa*); *Daly et al. 9932* (NY), March 1999 (*G. weberbaueri*); *Krukoff 5235* (NY), July 1933 (*G. sarcocarpa*); field observation, flowered in 1988 (*G. sarcocarpa*); *Daly et al. 12144* (NY), October 2003 (*G. sarcocarpa*).

Excluding deforestation up to 2001, we found the bamboo-dominated forests of the southwest Amazon cover 161,500 km^2^. Figure 5 shows this extent, along with the sites of all herbarium collections of flowering or fruiting bamboo, the location of the area (shown in Figure 3) where spectral analysis was conducted and the area of the life cycle study. At a site where adult bamboo density is low, e.g. herbarium collection *Daly et al. 12144,* (NY), bamboo was not detected in the two Landsat mosaics used for mapping. But the subtle change in reflectance between mature and post-mortality bamboo stages was detected here in a time series of TM 3-4-5 color composites. The bamboo-dominated forest extent is still underestimated in Figure 5. The two mosaics used for mapping have a temporal separation of less than ten years in some areas, so that some populations were at the juvenile stage and hidden in the understory in both mosaics.

The bamboo province is mostly on an eroded washboard relief of low amplitude with closely and regularly spaced hills. Along its northern edge, the bamboo province abuts dissected infertile argisols [11]. At its northeast edge, the bamboo *Guadua sarcocarpa* is excluded from low plateaus and their dissected remnants, with infertile leached latosol with sandy clay loam texture (eastern extremity of transect in Figures 3 and 4). Two sets of soil samples straddling this border show soil with bamboo to be a silty contractile clay richer in the exchangeable cations calcium, magnesium, sodium and potassium by one to three orders of magnitude (Table 1), compared to the nearby bamboo free latosol plateau. In some soil pits under dense bamboo, shrinkage in the dry season forms cracks, allowing organic matter to penetrate and darken the soil to a depth of 30 cm.

**Table 1:** Soil attributes compared between two transects along asterisks in Figure 3.

Soil attribute	Bamboo present (n = 7)	Bamboo excluded (n = 8)
Sand (%)	**5.7** (4.3)	**58.0** (11.0)
Silt (%)	**40.5** (5.3)	**15.6** (3.4)
Clay (%)	**53.1** (3.9)	**26.4** (11.3)
Shrinkage: change in specific volume on drying (Δml g^−1^)	**0.98** (0.30)	**0.16** (0.12)
Ca++ (cmol_c_ kg^−1^)	**20.4** (5.9)	**0.01** (0.02)
Mg++ (cmol_c_ kg^−1^)	**9.1** (4.5)	**0.39** (0.12)
Na+ (cmol_c_ kg^−1^)	**0.3** (0.1)	**0.003** (0.009)
K+ (cmol_c_ kg^−1^)	**0.16** (0.03)	**0.02** (0.01)
pH	**5.5** (0.2)	**4.3** (0.1)

Bold values are averages, one standard deviation in parentheses.

Means of samples taken at 85 cm depth. Each attribute’s mean is significantly different between the two transects (p = 0.001).

**Figure 6:**
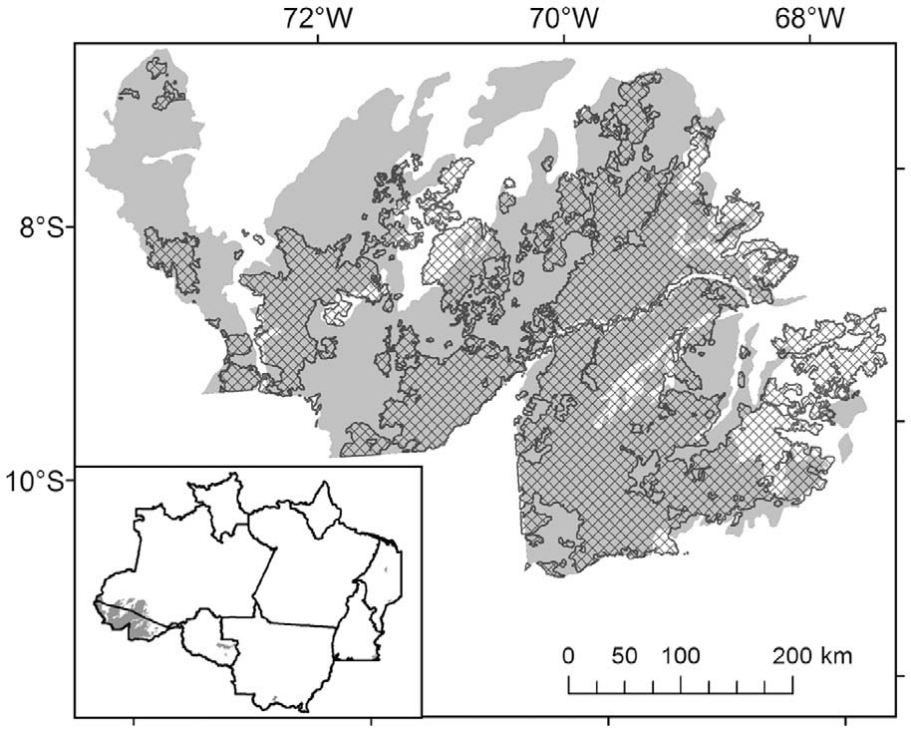
Bamboo-dominated forest (cross-hatch) coincides with eutrophic haplic cambisol and luvisol soils (gray). Inset shows these two fertile soil types are uncommon elsewhere in the Brazilian Amazon. White areas in Brazil on the main map are infertile argisols on dissected surfaces, gleysols along rivers and infertile latosol on low plateaus. Soil map from IBGE and EMBRAPA.

The same association between bamboo and fertile soil is seen at a larger scale. The most recent national soil map [11], which uses the Brazilian soil classification system [12], shows luvisols and eutrophic haplic cambisols covering 141,000 km^2^ of the southwest Brazilian Amazon, coincident with the bamboo-dominated forests (Figure 6). These two soil types are rare elsewhere in the Brazilian Amazon (Figure 6, inset).

### Life Cycle Length

**Figure 7:**
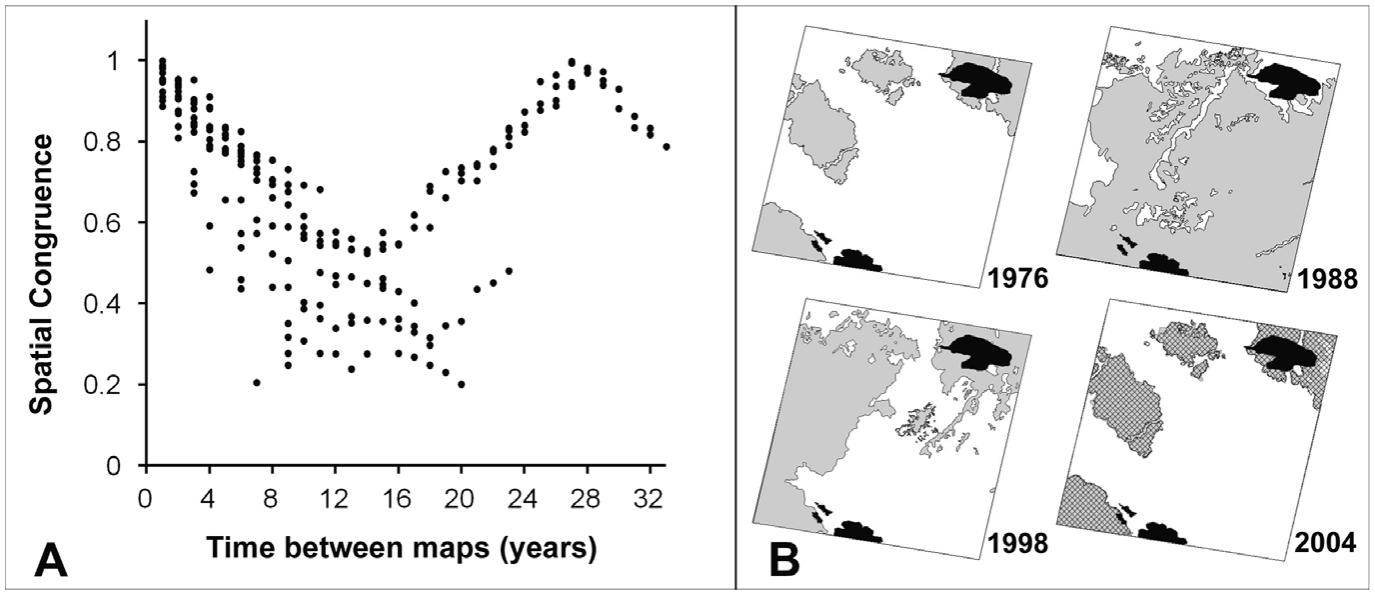
Bamboo life cycle of 27–28 y inferred from spatial congruence of mature-stage maps. (A) Congruence as a function of temporal separation. Each point represents one pair of mapped years of the 33,100 km^2^ study area. (B) Four of the mapped years spanning one full life cycle. Overlying the 2004 map is a hachure showing highly congruent extent of mature bamboo 28 y earlier. Gray is forest with mature bamboo at time of mapping, white is forest with juvenile bamboo hidden in understory, black areas were masked due to clouds.

Bamboo populations at the mature stage were mapped for 21 different dates spanning 33 y (Table 2), within the white square outline of 33,100 km^2^ shown in Figure 5. Spatial congruence between all pairs of maps approached 1.0, i.e., near-perfect agreement, when temporal separation reached 27–28 y (Figure 7A), which is therefore the inferred life-cycle length. Congruence declined for temporal separations exceeding 28 y. A near-perfect match was found between the 1976 and 2004 maps (Figure 7B). Any two maps spaced one life-cycle apart in time should match up, since each population that was hidden in the understory as juveniles or was visible in the upper canopy as adults in the first map of the pair will have returned to this same stage in the second map. This will only be true if all populations have about the same life-cycle length. For temporal distances of about one-half of a life cycle, spatial congruence was low and variable (0.2–0.6). Variability was caused by the large size of some bamboo populations. When these experienced a state change (adult to dead+hidden seedlings; hidden juvenile to visible adult), there was a large and abrupt shift in the spatial configuration.

**Table 2:** Dates and orbital sensors used for estimating bamboo life cycle length.

Year	Julian Days	Sensor
1975	202	Landsat MSS
1976	187	Landsat MSS
1979	172	Landsat MSS
1981	215	Landsat MSS
1985, 1986	197, 216	Landsat 5 TM
1988	231	Landsat 5 TM
1990	236	Landsat 5 TM
1991	287	Landsat 5 TM
1994	199	Landsat 5 TM
1996	205	Landsat 5 TM
1997	191	Landsat 5 TM
1998	194	Landsat 5 TM
2000	207	Landsat 7 ETM
2001	209	MODIS
2002	209	MODIS
2003	209	MODIS
2004	209	MODIS
2005	209	MODIS
2006	200	Landsat 5 TM
2007	187	Landsat 5 TM
2008	158	Landsat 5 TM

B-G-R color composites were made from Landsat Multi-Spectral Scanner bands equivalent to TM 2-3-4, Landsat Thematic Mapper bands 3-4-5 and MODIS bands equivalent to TM 3-4-5. MODIS data was from product 43B4, reflectance corrected to nadir view and illumination angles.

A life cycle of 27–28 y for all bamboo populations in this one 33,100 km^2^ area was supported by direct observation of three patches that experienced two consecutive post-reproductive mortality events. Their cycles were 27, 27 and 28 y. A fourth patch 50 km east of the 33,100 km^2^ area repeated its mortality after 32 y. Eighty km north of the study area, at the site of a flowering herbarium specimen of *Guadua scarcocarpa* collected in 1933 by botanist Boris Krukoff (08° 27′ S, 69° 52′ W; Figure 5), the bamboo population was seen to die in the year 1989 using Landsat images. This represents two full 28 y life cycles. We expect this patch to flower and die again in the year 2017.

### Clustering of Reproductive Events

All borders between two adjacent bamboo populations are sharply defined and clearly visible in Landsat TM and MODIS false-color composites, but only during and shortly after synchronous mortality of one of the two populations. During dieback of one population, there is no gradient in the spectral pattern close to the contact with the adjacent population. Therefore pixels near such a border are dominated by individual bamboo genets that are synchronized to one or to the other of the adjacent populations. A high degree of synchrony was also detected in a pair of Landsat 7 images spaced just 16 days apart (22 April to 08 May 2012; path 3, row 66). In the first image of this pair the border between two mature bamboo populations was not yet visible. In the second image the border was very distinct as the genets in one of the two populations all began their post-flowering senescence. Unanimous synchrony of all bamboo genets within a population was also supported by the 150 km over-flight of populations in the juvenile stage. No adults surviving from the previous generation were observed.

**Figure 8:**
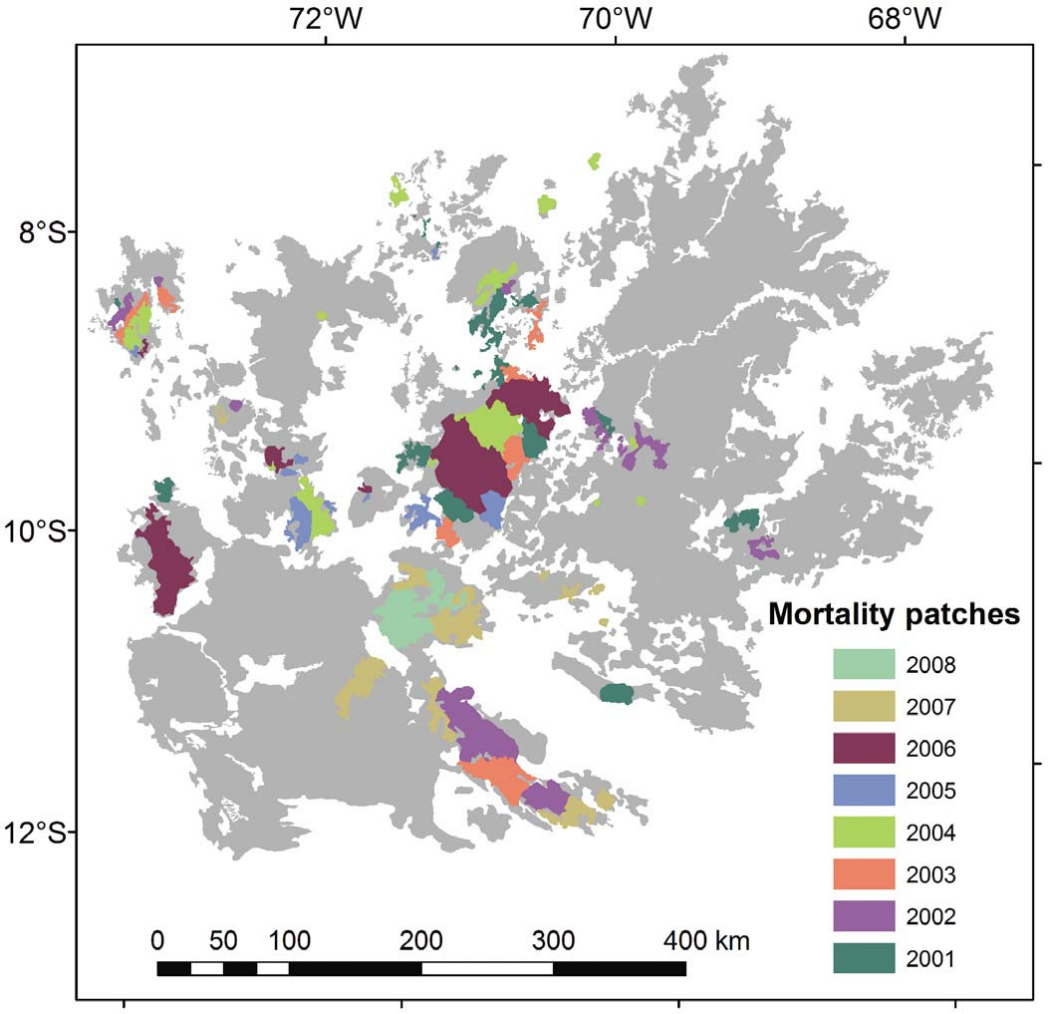
Spatial-temporal clustering of flowering events. Limits of 74 populations were detected in eight annual MODIS images (2001–2008). Gray area is full extent of bamboo-dominated forest in the southwest Amazon.

Over the entire area of the bamboo-dominated forests, 74 bamboo populations were identified and mapped using eight annual MODIS images (2001–2008). Each of these was mapped at the time of its internal synchronous mortality which follows immediately after flowering (Figure 8). The populations were spatially exclusive. The largest population covered 2,570 km^2^ and average size was 330 km^2^. Eight consecutive annual images constitute a temporally clustered set of observations spanning just 30% of the 27–28 y life cycle. Under the hypothesis of independent and random timing of flowering events, these 74 patches should be randomly distributed across the area of occurrence of southwest Amazon bamboo-dominated forests. They should also cover about 30% of the entire southwest Amazon bamboo area.

In fact, the 74 bamboo populations that flowered in the 8 y study period were spatially aggregated. They had more shared edge length than expected if these 74 patches were randomly distributed across the bamboo forest domain (p = 0.02). Visual inspection of Figure 8 also evinces a clustered distribution. Two-thirds of the flowering patches were adjacent to at least one other patch that flowered in another year between 2001 and 2008. These occupied 81% of the area of reproducing bamboos. Over half of the flowering patches, covering 76% of the total area of flowering bamboo in the eight years, had direct or contagious contact with two or more other patches that flowered. This spatial aggregation occurred despite each patch occupying, on average, only 0.2% of the total area of bamboo-dominated forests.

Total area of flowering/dying bamboo over the eight years was only 15% of the total extent of southwest Amazon bamboos, half the area expected. Temporally and spatially contagious flowering events formed seven large super-patches over the eight years. Only one of these contained a unidirectional flowering wave, which propagated from north to south. At least one other such flowering wave had, however, occurred earlier in the southwest Amazon, spreading from south to north [4].

## Discussion

### Spectral Patterns

In this study, the green vegetation fraction of forest with mature bamboo was only slightly different from that of primary forest without bamboo. The procedure used here for spectral unmixing employs a spectral library of many leafy forest types – ranging from bright secondary forest to darker and more textured primary forests – from which an optimum pure green vegetation spectrum is chosen for unmixing. This selection is made independently for each pixel, effectively ignoring differences due to canopy texture and associated shade/shadow. Shade is not used as a pure end-member. Both bright and dark forest canopies are treated as having high content of pure leafy vegetation.

If bamboo is very abundant it leaves larger gaps in the forest after dying. These are filled within two years by fast-growing pioneer trees [7], resulting in a spectral pattern similar to forest with mature bamboo in the canopy. Another source of confusion for detecting bamboo life stages arises in the dry season in forest with hidden juvenile bamboos. Many of the upper canopy trees become deciduous at the peak of the dry season, when most cloud-free satellite images are obtained. This increases the amount of exposed illuminated tree branches, emulating forest with recently dead bamboo. Both ambiguities are resolved by examining a longer time series of annual images and by including images from the earlier months of the dry season.

### Tectonic and Edaphic Control of Bamboo Distribution

The map of southwest Amazon bamboo-dominated forest coincides with the radially-shaped crustal bulge of the Fitzcarrald Arch (Figure 5, insets). Dense bamboo stops abruptly at the borders of this region which has distinct geomorphological, hydrological and soil properties. The arch is higher than surrounding terrain except where it abuts the Andes. It is flanked to the northwest, east and southeast by basins with flat relief [13], [14], free of bamboo. Seventh-order river basins on the arch have immature shape and hypsometry, indicating active or recent uplift [15]. Its peculiar high-frequency, low-amplitude washboard relief is not found over any other area of comparable size in the Brazilian Amazon.

Our soil collections are only a case-study, limited to a small portion of the edge of the bamboo-dominated forest. Nonetheless, characteristics of the bamboo-dominated soils studied there were consistent with those of luvisols and eutrophic cambisols, which are clearly associated with bamboo across the rest of the Brazilian southwest Amazon (Figure 6). Both these soil types have high base saturation. Luvisol B-horizon clays have high cation exchange capacity, generally being 2∶1 clay minerals which expand when wetted and shrink and crack upon drying [12].

Four additional lines of evidence indicate that the substrate across the bamboo province is easily eroded, poorly drained and rich in cations. First, Landsat images show that streams and minor tributaries arising throughout the bamboo province are rich in suspended sediment, even in forested landscapes and despite the low amplitude of relief within low-order basins. This indicates a high rate of mechanical erosion. Second, small streams dry up completely in the dry season, suggesting incomplete recharge of ground water in the rainy season, due to low permeability of the substrate. Third, all rivers draining the bamboo province which have been studied to date have high conductivity: 310, 190 and 270 µS.cm^−1^ in the Iaco, Caeté and Chandless Rivers [16], and 390 µS.cm ^−1^ on average in tributaries of the upper Acre River [17]. These conductivities are much higher than central Amazon streams draining infertile upland. This also indicates that mechanical erosion has occurred, exposing cation-rich Miocene tidal sediments or bringing them close to the soil surface [18]. Finally, fertile but poorly drained soils explain why several tree species found associated with bamboo on upland in the southwest Amazon are elsewhere confined to floodplains of muddy rivers fertilized by sediment from the Andes. Examples are *Couroupita* sp., *Ceiba pentandra*, *Calycophyllum* sp., *Hura crepitans* and *Triplaris* spp. This all suggests that low hydraulic conductivity shunts rainfall into surface runoff during the rainy season, allowing mechanical erosion to continue apace with uplift of the Fitzcarrald arch, leading to shallow soils and providing a fresh supply of unleached fertile parent material from below.

The match between bamboo limits and soil type is also evident in the small scale Amazon soil map of Quesada *et al.* [19] The northern edge of bamboo is approximately coincident with their Cambisol/Acrisol border, using FAO soil classes. Relatively fertile soils can be found elsewhere in the western Amazon, for example, where the highly leached Nauta and Iça formations have been removed to expose cation-rich estuarine sediments of the Pebas Formation [20]. However, sarmentose bamboos are not dominant on dissected upland soils away from the Fitzcarrald Arch.

### Life Cycle Length

The estimate of life cycle length using spatial congruence of adult stages, points to only minor variation within the study area of 33,100 km^2^. If life cycles were more variable, congruence between maps would never approach 1.0. Episodes of reproduction and mortality occurred throughout the entire 33 y study period, so environmental cues such as unusually wet or dry years are not implicated as triggers for flowering events. This indicates that the life cycle is genetically programmed. One population of *Guadua sarcocarpa* 80 km north of the study area had a similar life cycle length (28 y), but an unidentified population 50 km to the east showed a 32 y cycle. This difference may be a consequence of having at least two closely related sarmentose *Guadua* species in the southwest Amazon. These cannot easily be distinguished in the field when lacking flowers or fruits [21] and probably cannot be separated by remote sensing.

### Flowering Waves: Types, Origins and Historical Biogeographic Implications

In his discussion of the literature on gregariously flowering bamboos at large spatial scales, Franklin [22] described three types of temporal-spatial organization of the phenomenon: (1) imperfectly synchronous but time-centered flowering of individuals within a single population; (2) internal synchrony of each population with semi-synchrony between distinct populations, constituting flowering waves; and (3) variation in the life-cycle length between populations. Flowering waves are always temporally organized, but are not necessarily spatially organized.

From Landsat images it is not possible to detect a few individuals that flower and die a year early or late within a dying adult cohort of bamboo. Nonetheless, each dead patch exhibited a homogeneous color on any single Landsat image. Because the spectral response of the canopy containing dead bamboo changes quickly as the dead branches rot and fall to the forest floor, this spatial homogeneity indicates that (1) there is no migration of the flowering time across the internal area of a single population and (2) there is a strong reproductive synchrony within a population.

Distinct populations of the sarmentose *Guadua* species of the southwest Amazon show both spatial and temporal clustering of reproduction (Franklin’s type 2 above), here called super-patches. Only 19% of all flowering bamboo area over the eight-year study was found outside the super-patches. Most flowering waves spread across a super-patch in a complex fashion, not by unidirectional contagion.

Are flowering waves consistent with the hypothesis of widespread bamboo invasion in the southwest Amazon, abetted by the disturbances of indigenous swidden agriculture and associated forest ground fires [23]? Whatever the mechanism for establishing the close timing of reproduction in spatially adjacent populations, it would require a long history of *in situ* evolution since each population reproduces only once in 27–28 years. Four understory bird species are obligate specialists for *Guadua weberbaueri* thickets in the Peruvian Amazon [24]. Nine other bird species are near-obligates. Most of these 13 species have ranges limited to the southwest Amazon, while non-bamboo specialists have wider ranges. This also points to a very long history of widespread bamboo in the SW Amazon.

Griscom & Ashton [1] also argued in favor of a non-anthropogenic origin for these forests, showing that the adult bamboo itself provides sufficient disturbance of trees to maintain high density. Bamboos of the southwest Amazon are favored by canopy openings left by fire [9] and are very resilient after fire [10]. But fire does not occur every life cycle upon death of the adult cohort, as required by the bamboo-fire hypothesis of Keeley and Bond [25]. Bamboo itself, not fire, appears to be the main source of disturbance which opens canopy gaps colonized by dense thickets of bamboo [1], [9]. Even without the disturbance to trees provided by the mature bamboo, and without fire, ten years after these adults have died the juvenile bamboos arising from seed are able to regain dominance of the forest canopy.

Franklin [22] hypothesized that the temporal clustering of flowering events between neighboring populations of a woody riparian bamboo in Australia – and of a woody bamboo in India [26] – is a product of the allochronic divergence of daughter populations derived from a single ancestral population. A small group of closely spaced individuals could become offset in the same temporal direction by stochastic mechanisms, without spatial separation or environmental triggers. Stochastic allochronic divergence by individuals spatially contained within the mother population can explain very small daughter populations. Expansion of such a small group of individuals into the population sizes seen in the southwest Amazon would require that they have some competitive advantage over the sympatric mother population from which they were derived.

There is evidence from satellite images suggesting this may have occurred. One case shows clear partitioning of the upland into two topographic niches occupied by two different bamboo populations or species. At another border between neighboring populations or species, the contact is jagged and outliers of one of the two populations persists as very small remnants within a matrix of the other. This suggests expansion of the matrix population into the former territory of its neighbor.

A second possible mechanism for attaining the small temporal offsets in flowering times seen in the super-patches is evident at some edges between bamboo-dominated and bamboo-free forest. Where these edges are not associated with a change in landform, they are rounded. Furthermore, perfectly round outlier patches of bamboo lie within 10 km of the main frontier of the presumed source population [9]. Rounded borders indicate that semi-scandent *Guadua* has not yet fully occupied the distinct cation-rich soils overlying the Fitzcarrald Arch. Colonization of suitable substrate is still proceeding and outliers are only temporarily separated in space from one another and from their expanding mother population. Over many generations, this spatial separation may allow a slight drift in flowering time. If just a few dispersed seeds were the successful founders of an outlier population, these can also be stochastically offset in the same temporal direction from the start. (Though comprising a small percentage of a population, individuals that flower one year before or after the main flowering within a single a woody bamboo population have been described for other woody bamboos [22], [27].) When vegetative expansion finally brings the outliers back into contact with one another and with the mother population, they could maintain their slight asynchrony, forming the spatially and temporally clustered components of super-patches.

Periodic cicadas in North America provide a model for the origin and evolution of bamboo flowering waves by incipient allochronic speciation [22], [28], [29]. Each cicada species is divided into year classes. Each year class reproduces synchronously at the end of a 13y or a 17y cycle. Similar to southwest Amazon bamboos, the cicada populations belonging to a single year class of a species occupy large patches that are temporally isolated from and are parapatric with the patches of other year classes of that species. Also like bamboos, those patches which reproduce in succeeding years are often spatially adjacent to one another [30].

### Conclusions and Future Directions

1. At least 161,500 km^2^ of the southwest Amazon is occupied by forest with high density of sarmentose woody bamboos of the genus *Guadua*.
2. Forest with mature bamboo, with recently dead bamboo and with juveniles confined to the sub-canopy (or lacking bamboo) are easily distinguished in false-color composites of orbital sensors employing a visible, a near-infrared and a short-wave infrared band. These stages can also be distinguished with the older MSS sensor despite lacking a short-wave infrared band.
3. Greenness varies only slightly between these three forest types, indicating that “bamboo-dominated” forest canopies are actually dominated by evergreen trees.
4. The southwest Amazon bamboo province is associated with an area of tectonic uplift with high rates of mechanical erosion. This has decreased soil thickness, bringing close to the surface parent sediments of low hydraulic conductivity, rich in cations.
5. The bamboo life cycle is about 27–28 y in length.
6. Each bamboo population averages 330 km^2^ in size and is reproductively synchronized internally. Many are semi-synchronized with neighboring populations, causing flowering waves.
7. It is now possible to predict where and when new episodes of bamboo mortality will occur. This is of practical value for forest management and social relief planning. Falling culms of dead spiny bamboo make it difficult to maintain trails through the forest. Where bamboo density is high, rubber tappers are forced to abandon their trade during the year of mortality.
8. Spatial patterns of landscape occupation and temporal patterns of flowering raise testable hypotheses. Are semi-synchronized clusters comprised of incipient species resulting from recent allochronic divergence? Is one bamboo species replacing another at the few jagged borders between adjacent populations?
9. Spatially and temporally organized patterns of monocarpic bamboo reproduction have probably evolved over a long time, militating against the hypothesis of a bamboo invasion of the southwest Amazon, facilitated by the disturbances of swidden agriculture or human-induced ground fires.

## Materials and Methods

Basic procedures are summarized in this paragraph then described in greater detail. Spectral patterns of bamboo life stages, spectral separability of these life stages and mapping of adult stage bamboo were all initially guided by analysis of a single Landsat image which contained forest without bamboo in the upper canopy and forests with mature and with recently dead bamboo. Surface reflectance was retrieved from this image using CLASlite [31]. The three forest types were then sampled to train linear discriminant analysis and obtain average Bhattacharyya distances to measure how well these three forest types can be discriminated using different three-band color compositions available from other orbital sensor products covering the entire southwest Amazon, with dates going back as far as 1975. CLASlite was also used to run a mixing model to provide a physical explanation for the spectral changes associated with dead bamboo.

### Spectral Pattern of Bamboo Life-stages

To compare the spectra of forests with mature bamboo, recently dead bamboo and no bamboo in their upper canopies, we used atmosphere-free reflectance values of the six optical bands of a Landsat TM image. To eliminate inter-date errors in atmosphere removal and sensor degradation, all spectral data analyzed in this paper were from this one image (World Reference System 2, path 2, row 66, July 26, 1988). Samples were further constrained to a 40 km wide area in the east-west direction, reducing variation in view and solar zenith angles to 3° and 0.4°, respectively. These are sufficiently narrow to eliminate artifacts caused by view and illumination geometry[32]–[34].

**Figure 9:**
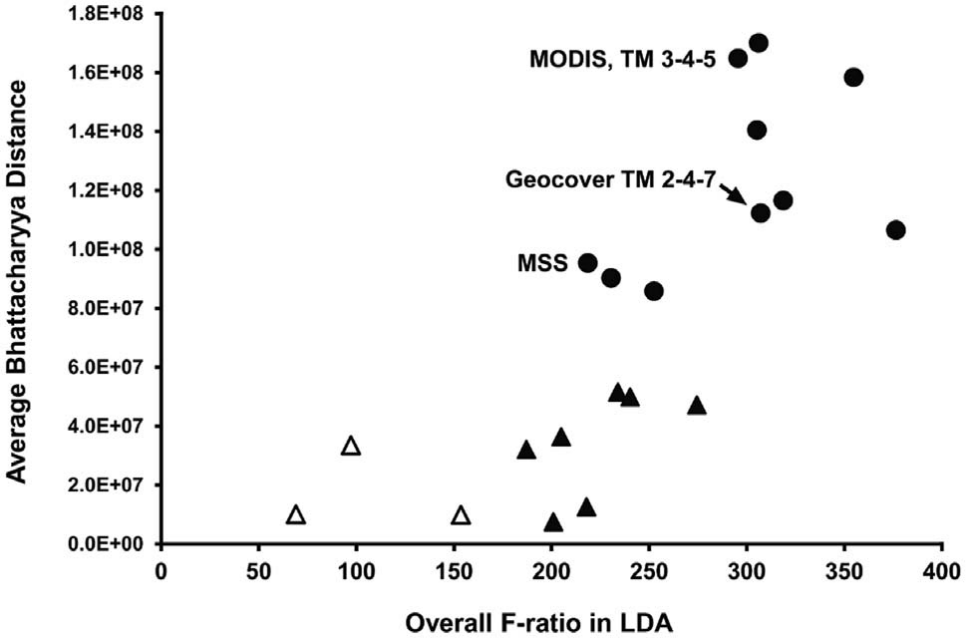
Performance of three-band combinations for distinguishing mature bamboo, dead bamboo and no bamboo in upper canopy. Equivalent Landsat TM bands were used to represent MSS and MODIS bands. Higher values of B-distance and of F-ratio indicate better target separation in false-color composites. Using a leave-one-out jackknife validation, 17 of the 20 combinations correctly classified 100% of all samples (solid symbols). Better combinations all include a near-infrared band (circles). Labels indicate false-color composites from sensors used in this study: Landsat MSS equivalent to TM 2-3-4 (maps for life cycle estimation); Landsat Geocover TM 2-4-7 (map of bamboo extent); Landsat TM 3-4-5 (life cycle) and MODIS equivalent to TM 3-4-5 (life cycle, bamboo extent, mortality patches).

We used linear discriminant analysis (LDA) to determine whether the three spectra are distinct. We used both LDA and Bhattacharyya distance (B-distance) to determine which combinations of bands provide good separation of the three forest types in false-color composites (Figure 9). We were interested in the combination TM 2-4-7 of the Landsat Geocover mosaics for mapping the full extent of mature bamboo; the equivalent to TM 2-3-4 from the Landsat MSS sensor, required for dates prior to 1985 in the life cycle study; and the standard TM 3-4-5 or its equivalent, used for all other Landsat and MODIS images in this study. The MSS band combination performed well (Figure 9), despite the lack of a short-wave infrared band.

Bamboo-dominated forests at the mature, recently dead (post-flowering) and juvenile stages, and a forest without bamboo were all visited in the field in the region of the 1988 image. At four other sites across the southwest Amazon, we used the dates and locations of herbarium collections of flowering or fruiting specimens of *Guadua sarcocarpa* and *G. weberbaueri* as field evidence of the life stage to confirm and extend observations of spectral changes. We examined pre- and post-flowering TM 3-4-5 color composites at these four sites. Two low-altitude over-flights were made, one over a population of fruiting/senescing *Guadua sarcocarpa*, the other over 150 km of *Guadua* sp. at the juvenile stage, confined to the forest understory. All field visits were on private land with permission from the owners.

We used Tukey post-hoc pairwise comparison to detect differences in greenness of the three forest types in the 1988 test image. Canopy greenness indicators were the green vegetation fraction (illuminated and partially shaded leaves) and the non-photosynthetic vegetation fraction per pixel (dead leaves, bare wood, bare branches) obtained from linear spectral unmixing. Spectra, LDA, B-distance and greenness comparisons were all based on 20 square samples of 100 pixels from each the three forest types in the 1988 image, i.e. 2000 pixels per type.

Discriminant analysis and means comparisons were conducted with Systat v. 9. Bhattacharyya distances were obtained with Idrisi v. 17. Processing to atmosphere-free reflectance and the linear unmixing of pixel fractions were performed with CLASlite v. 2 [31]. On a pixel-by-pixel basis, this program selects the best combination of pure end-members from a library of three spectral bundles, one for pure non-photosynthetic vegetation (NPV; i.e. bare bark, dead wood, dead leaves), one for pure green vegetation and one for pure bare soil. Shade is not included as a potential end-member since each of the three bundles includes pure spectra ranging from dark to light. The end-member spectra for pure green vegetation were obtained from above the forest canopy using the Hyperion orbital sensor. This data was from the Brazilian and Peruvian Amazon, Costa Rica and Hawaii. The pure soil and pure NPV spectra were obtained using field spectrometers at ground level in Amazonia, Central America and Hawaii [31].

### Extent of Bamboo Dominated Forest in the Southwest Amazon

We mapped all bamboo-dominated forests with bamboo at the mature stage, inside a 475,000 km^2^ rectangle bounded by 6.5–12.5 degrees of south latitude and 67.5–74.0 degrees of west longitude. All mapping was by visual interpretation at a scale of ∼1∶250,000. To capture almost all bamboo populations at the visible mature stage we used the Landsat Geocover mosaics (TM bands 2-4-7) spaced about ten years apart, dated ∼1990 and ∼2000. The year ∼2000 mosaic has many clouds. To fill these data gaps, we used a single cloud-free 16-day temporal and spatial mosaic of MODIS images covering the entire study area, acquired in July of 2001. Bands equivalent to Landsat TM 3-4-5 were chosen. The MODIS reflectance values are corrected for effects of atmosphere and for variations in reflectance associated with different combinations of view angle and sun angle [35]. Pixel size is 1×1 km. Areas deforested as of 2001 were masked in the final map.

On both sides of a border where the edge of bamboo forest is clearly defined by landform (Figure 3), we also compared soil texture, fertility and soil shrinkage on drying. Samples were distributed along two transects on either side of this border and collected at 85 cm depth. By sampling at this depth where there is little organic matter, we compared soil nutrients (i.e. <200 cm depth) derived from the soil parent material rather than nutrients derived from forest biomass that are simply being recycled back to the forest. Soil maps and soil type terminology follow the Brazilian soil classification system [12].

### Length of the Bamboo Life Cycle

From the three different orbital sensors, Landsat MSS, Landsat TM and MODIS, we obtained images for 21 dates spanning 33 y (Table 2). At each date, we mapped all areas with bamboo at the mature stage, based on visual interpretation of color composites using bands that had provided strong separation of bamboo life stages in the 1988 test image (Figure 9). Mapping scale was ∼1∶250,000. We then compared the spatial congruence of all pairs of the 21 maps. This was conducted within the geographic area of a single Landsat scene (World Reference System 1, path 3, row 67, covering 33,100 km^2^ after masking clouds) far from roads or other deforestation fronts. If all bamboo populations in the study area have about the same life cycle length, any two maps spaced exactly one life cycle apart in time should show high spatial similarity. The adult stage is actually a long period of time within the life cycle of a population so each mapped polygon of the adult stage includes several contiguous populations which will flower and die in different years. Nonetheless, the precision of this inferential method will be high if one or more bamboo populations experience a detectable state change (adult to dead; juvenile to adult) every year or two.

### Spatial-temporal Clustering of Reproducing Populations

The evolutionary pressure on individuals at the sharp border between two distinct monocarpic bamboo populations to “choose” between the two life cycles in order to guarantee pollination should result in neighboring populations that exchange little or no pollen. Without gene exchange, one would expect that the flowering of a population should occur independently from the timing of its neighbor populations. To test this hypothesis, we mapped the extent and date of all populations experiencing post-reproductive mortality. Mortality is a good proxy for the one-time flowering of each population patch, because senescence begins when the bamboos are still laden with fruit. We did this over the entire range of the southwest Amazon bamboos, using eight annual MODIS mosaics obtained in July of each year from 2001 to 2008.

We tested for spatial aggregation of the patches (i.e. populations) which flowered in the eight year period by measuring the total shareable edge length of all flowering patches. We determined what fraction of this edge length was in fact shared. Shareable edge length excludes those portions of the patch edge which coincide with the border of the bamboo forest domain. We determined the probability of the observed amount or more of shared edge occurring under a random spatial allocation of the flowering population patches within the entire domain. For this we created 20 model domains, each with a random distribution of flowering patches. We measured the shared fraction of shareable flowering patch edge length in each of these domains, to obtain the probability distribution (average and standard deviation) of this attribute for the random distribution of flowering patches. As in the real world over the eight year period, each of the 20 domains had 15.3% of its area occupied by 74 flowering population patches. The remaining 84.7% of the area was occupied by 410 non-flowering patches. In the real world we do not know the number, size and position of these non-flowering patches. So, to permit randomized spatial distributions, all flowering and all non-flowering simulated patches were of the same size, being the average size of the 74 known patches. Simulated patches were square in shape. Patches with irregular shapes would not fit together when randomized. The square shape is justifiable considering that, in the real world, all neighboring bamboo populations behave like square cells in a matrix, since they always fit together along their adjacent borders despite having irregular shapes. To account for the convoluted perimeter and the holes in the entire bamboo forest domain, which affect the amount of shareable patch edge, we produced two sets of 20 domains. The first set consisted of 20 random allocations of 74 flowering patches inside a square 22x22 matrix of all bamboo population patches. This configuration has a high total shareable patch edge length, because most flowering patches will not be along the border of a square domain. The second set consisted of 20 random allocations of the 74 flowering patches inside an elongate 1x484 arrangement of all bamboo patches so that all flowering patches have two non-shareable sides along the domain border. This represents a highly convoluted bamboo forest domain. In the results we report the more conservative of the two p-values derived from these two sets of modeled domains.
